# The ovarian cancer-associated microbiome contributes to the tumor’s inflammatory microenvironment

**DOI:** 10.3389/fcimb.2024.1440742

**Published:** 2024-10-21

**Authors:** Min Zhang, Jiahang Mo, Wu Huang, Yiting Bao, Xukai Luo, Lei Yuan

**Affiliations:** ^1^ Department of Gynecology, Obstetrics and Gynecology Hospital, Fudan University, Shanghai, China; ^2^ Institute of Reproduction and Development, Obstetrics and Gynecology Hospital, Fudan University, Shanghai, China

**Keywords:** ovarian cancer, microbiome, inflammatory microenvironment, bacteria, immune cells

## Abstract

A growing body of research has established a correlation between tumors and persistent chronic inflammatory infiltration. As a primary instigator of inflammation, the majority of microbiomes naturally residing within our bodies engage in a mutually beneficial symbiotic relationship. Nevertheless, alterations in the microbiome's composition or breaches in the normal barrier function can disrupt the internal environment's homeostasis, potentially leading to the development and progression of various diseases, including tumors. The investigation of tumor-related microbiomes has contributed to a deeper understanding of their role in tumorigenesis. This review offers a comprehensive overview of the microbiome alterations and the associated inflammatory changes in ovarian cancer. It may aid in advancing research to elucidate the mechanisms underlying the ovarian cancer-associated microbiome, providing potential theoretical support for the future development of microbiome-targeted antitumor therapies and early screening through convenient methods.

## 1 Introduction

Ovarian cancer (OC) is the prevalent malignancy within the female reproductive system, characterized by a high incidence and mortality rate (Siegel et al., 2023). As of 2022, OC accounted for an estimated 3.4% of new cancer cases and 4.8% of cancer deaths in women (Bray et al., 2024). Globally, there are annually over 320,000 new cases and 200,000 deaths attributed to OC (Bray et al., 2024). However, the early stages of OC often present with subtle or nonspecific symptoms, and the ovaries' deep abdominal location makes early palpation of small masses challenging, both of which contribute to the delayed diagnosis until advanced stages (González-Martín et al., 2023). Moreover, numerous clinical studies have demonstrated low positive rates of early imaging and serum screening for OC, further hindering early detection (Buys et al., 2011; Menon et al., 2021, 2015). Given the high recurrence rate and limited options for early detection, urgent advancements in research are imperative to improve OC prognosis. The refractory nature of OC has spurred an intensified investigation into its pathogenesis and the development of targeted therapies. While current guidelines suggest the potential efficacy of immune-related targeted drugs, the persistent high recurrence rate and poor response to treatment remain substantial challenges.

Inflammation is a fundamental pathological process that occurs in living tissues involving the disseminate of the vascular system. It is triggered by various harmful factors, which commonly invade the organism through physical, chemical, and biological means (Medzhitov, 2008). An increasing number of research has demonstrated a strong association between tumors and persistent inflammatory processes, such as obesity and ovulation in OC (Liu et al., 2015; Ness et al., 2000; Shea et al., 2023). The progression of tumors is considered to be associated with immune suppression, whereby inflammatory factors impede antitumor immunity and modify the tumor microenvironment (TME) (Greten and Grivennikov, 2019; Niu and Zhou, 2023). Infections caused by bacteria and viruses are primary instigators of inflammatory processes. In normal physiological conditions, the microbiome residing in areas such as the reproductive tract and the intestines interacts with the body in a reciprocal manner. Disruptions to this equilibrium can lead to the development of diseases including neoplastic growth. Approximately 20% of all human cancers are associated with chronic inflammation arising from persistent infections (Elinav et al., 2013). As widely recognized, *Helicobacter pylori* infection is related to gastric cancer, hepatitis B or C infection is linked to hepatocellular carcinoma, and human papillomavirus (HPV) infection is associated with cervical cancer (Hashimoto et al., 2018; Parkin et al., 2020). In addition to the tumor-associated microbiome mentioned above, recent studies have identified associations between tumors and the microbiome in various locations. *Clostridium* and its metabolite trimethylamine N-oxide have been shown to inhibit triple-negative breast cancer by activating the endoplasmic reticulum stress kinase PERK and thus enhancing CD8+ T cell-mediated antitumor immunity (Wang et al., 2022a). *F. nucleatum* can promote colorectal cancer through microbiome-derived formate by triggering aryl hydrocarbon receptor signaling (Ternes et al., 2022). Regarding OC, the intrinsic microbiome within the ovarian niche such as *Propionibacterium acnes*, the upward migration of pathogens, and even gut microbiome dysbiosis have been implicated in OC development (Hu et al., 2023; Huang et al., 2022a; Li et al., 2023). Therefore, this review will focus on the altered composition of the microbiome and associated inflammatory changes in specific sites, aiming to explore the microbiome's influence on the inflammatory microenvironment of tumors and provide insights for early diagnosis and precision therapy of OC.

## 2 Relationship between inflammation and ovarian cancer

**Figure 1:**
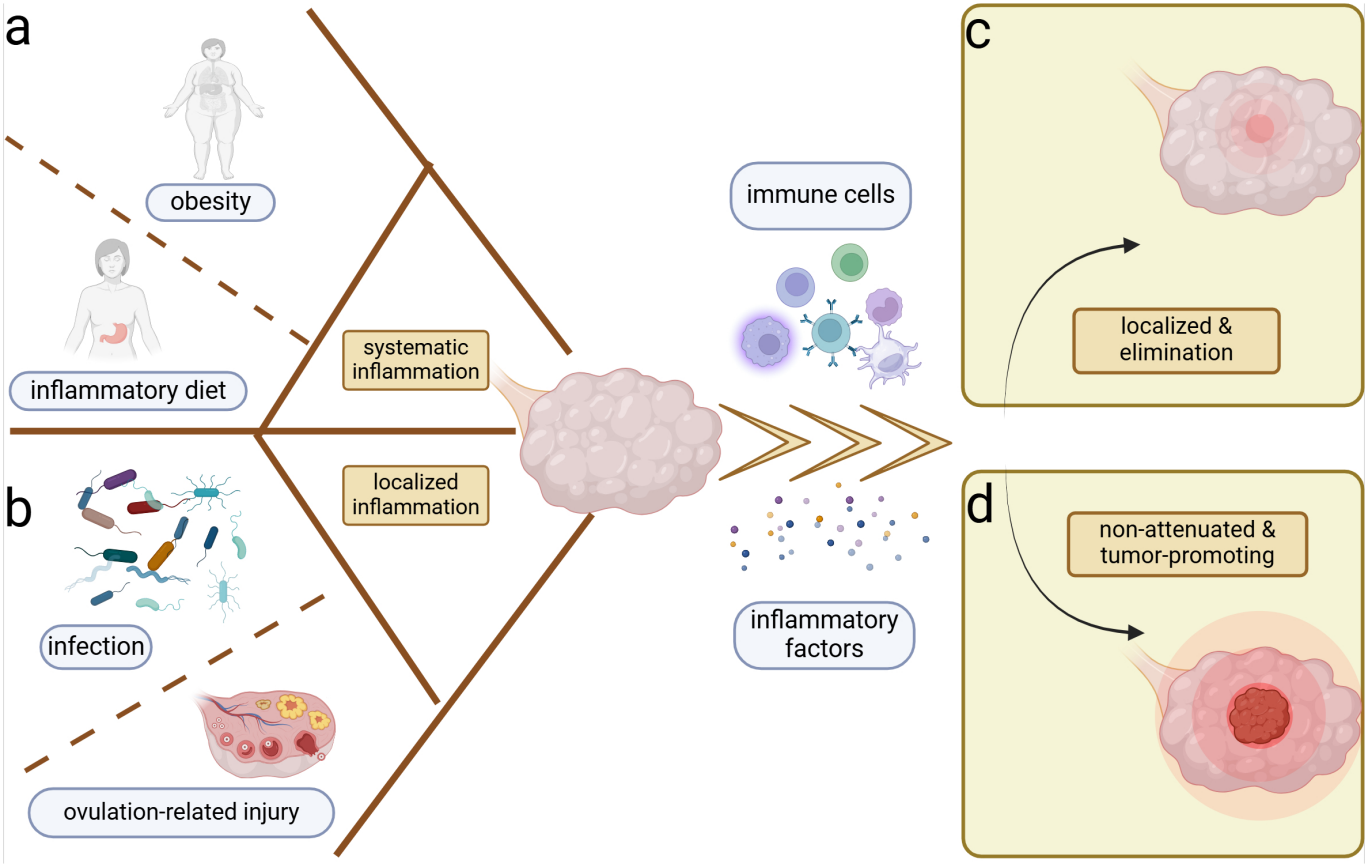
Inflammation and ovarian cancer. Systemic and local inflammation can both induce the ovarian inflammation and potential carcinogenesis. **(A)** Obesity and sustainable inflammatory diets are most important for the perpetual systemic inflammation. **(B)** Cyclic ovulation injuries and chronicity of microbiome infection can prompt the persistent localized inflammation of the ovary. Inflammation induces the accumulation of immune cells and inflammatory factors in the ovarian microenvironment, which yield two distinct outcomes. **(C)** Localization and elimination of inflammation–restoration of homeostasis. **(D)** Ongoing and non-attenuated inflammation–tumor promotion.

Systemic inflammatory conditions such as obesity (Shea et al., 2023) as well as localized inflammatory conditions such as ovulation (Ness et al., 2000) and infections (Paavonen et al., 2021) are associated with the development of OC (**Figure 1**). Typically, tissue injury induces the aggregation of immune cells and inflammatory factors to combat microbial invasion promoting the healing of the wound (Peña and Martin, 2024), which is followed by the subsidence of inflammation and the changes of immune cells and inflammatory factors to normal levels (**Figure 1C**). In contrast, tumors are considered as non-healing wounds (Dvorak, 2015). The persistent inflammatory stimulus promotes uncontrolled tumor cell proliferation and diminishes the damaging capacity of immune cells within the microenvironment (Schäfer and Werner, 2008). Ovulation is a continuous process of wound-healing and cyclic inflammatory stimulation, and studies have recognized its association with OC (Fathalla, 1971, 2016) (**Figure 1B**). The repair of minor ovulation trauma recruits a significant number of inflammatory factors, including prostaglandins, interleukins (ILs), and tumor necrosis factor-α (TNF-α) to the injured epithelial surface (Macciò and Madeddu, 2012), which can also alter the systemic inflammatory state. Obesity and inflammatory diets are associated with systemic inflammation, often characterized by Western-style diets with a high proportion of refined grains, fat, and processed meat, but low in unprocessed fruits, vegetables, and whole grains (Malesza et al., 2021; Shea et al., 2023) (**Figure 1A**). Both obesity and inflammatory diets can increase the risk of OC by perpetuating the local inflammatory microenvironment through elevated proinflammatory factors like IL-10 and the depletion of antitumor immune cells in the peritoneum (Liu et al., 2015; Shea et al., 2023).

In addition to physicochemical injury-induced inflammation, microbiomes are equally significant inducers of inflammation and have been implicated in OC development (**Figure 1B**). Body cavities that communicate with the external environment such as the genital and intestinal tracts are colonized by abundant commensal microbiomes, which help to establish the physicochemical and immune barriers, exemplified by the predominance of *Lactobacillus* in the vagina and the probiotics in the intestinal tract (Fu et al., 2023; Zhu et al., 2022). *Lactobacillus* aggregated in the female reproductive tract can form biofilms to prevent pathogenic bacteria adhesion (Zhu et al., 2022). Furthermore, they produce lactic acid, which increases the chemotaxis of anti-inflammatory factors like interleukin 1 receptor antagonist (IL-1RA) and inhibits the aggregation of pro-inflammatory factors like TNF-α (Zhu et al., 2022). Regarding the intestinal microbiome, the regulation of immune cell function contributes to homeostasis. For example, *Clostridium* promotes the accumulation of T regulatory cells (Tregs) (Atarashi et al., 2011), and *Akkermansia muciniphila* expands T follicular helper cells (Ansaldo et al., 2019), which help to stabilize the intestinal immune environment. Metabolites such as short-chain fatty acids can regulate the production of cytokines chemokine ligand 20 (CCL20) and IL-8, suppressing inflammatory responses within the tumor (de Vos et al., 2022; Fu et al., 2023; Shibata et al., 2017). When internal or external disturbances disrupt the commensal microbiome, pathogens can exploit the compromised homeostasis. During the body's resistance to pathogenic microbiomes, various mechanisms are employed to maintain homeostasis, including microbial phagocytosis, microbial lysis, and the elimination of microbial-associated toxins (Chaplin, 2010). However, under abnormal conditions, the pathogenic microbiome may not be completely eradicated. Chronic pelvic inflammatory disease and vaginal microbiological dysbiosis have been linked to ovarian carcinogenesis (Lin et al., 2011; Nené et al., 2019). The persistent presence of local inflammation without complete elimination is suspected to be associated with tumor development (Liu et al., 2022), potentially mirroring a similar process in OC (**Figure 1D**).

## 3 Ovarian cancer-associated microbiome

Microbiomes are abundant in various body regions, including the skin, oral mucosa, nasal mucosa, gastrointestinal mucosa, genital mucosa, and urinary tract. Naturally, they coexist with us harmoniously, contributing to internal homeostasis. However, the alterations in their abundance and composition can lead to tissue damage and even cancer development through toxic and inflammatory injury. Several studies have demonstrated that microbial alterations can induce phenotypic transformations of macrophages (Kostic et al., 2013; Xu et al., 2019) and dendritic cells (DCs) (van Teijlingen et al., 2020) as well as functional differentiation of T cells (He et al., 2021; Zhu et al., 2023a) and B cells (Ursin et al., 2022; Zorea et al., 2023). With increasing research confirming the presence of microbiomes in the upper reproductive tract (URT), their origin and pro- or antitumoral roles in OC are becoming a focal point of attention. This section concentrates on the immune-associated inflammatory changes that affect the TME.

### 3.1 Intratumor microbiome and ovarian cancer

Ultrasensitive genetic testing techniques such as 16S rRNA gene sequencing, Patho-Chip (pan-pathogen array), and 2bRAD sequencing have advanced the study of the intratumor microbiome (Banerjee et al., 2017; Wang et al., 2023; Wensel et al., 2022). Beyond genetic testing, the presence of bacteria in OC has also been confirmed through immunofluorescence of lipopolysaccharide (LPS) (Wang et al., 2020). Moreover, Nejman and colleagues have traced the presence of bacteria within immune cells and tumor cells, including OC, using diverse diversified visualization methods (Nejman et al., 2020).

In general, the microbiome diversity and richness within OC niches are diminished, with certain cultures becoming relatively more abundant compared to the non-cancerous tissue (Wang et al., 2020; Yu et al., 2024; Zhou et al., 2019). Regarding specific measurable changes in the microbiome, *P. acnes*, *Acetobacter*, *Firmicutes*, *Proteobacteria*, and *Fusobacterium* have shown relative increases, while *Lactococcus* has decreased (Banerjee et al., 2017; Huang et al., 2022a; Nejman et al., 2020; Wang et al., 2020; Yu et al., 2024; Zhou et al., 2019). Some of these bacteria have been demonstrated to induce local inflammatory microenvironment formation through inflammatory signaling pathways and oxidative stress responses. By isolating and culturing specific strains, Huang and colleagues validated the abundance of the aforementioned bacterial genera and identified *P. acnes* as the most prominent strain in OC (Huang et al., 2022a). Researchers also confirmed its tumor-promoting function in epithelial ovarian cancer (EOC), where it activates the Hedgehog pathway with increased inflammatory factors such as TNF-α and IL-1β (Huang et al., 2022a). Iron-induced oxidative stress and subsequent DNA mutations in clear-cell OC induced by *Acetobacter* and *Lactobacillus* contribute to the persistent inflammation, resulting in the activation of oncogenes that promote tumor progression (Kawahara et al., 2024). The bacterial-toxic metabolite LPS is associated with tumor progression. LPS can stimulate tumor cell progression by activating Toll-like receptor 4 (TLR4) through IL-6 (Kashani et al., 2020), which can subsequently induce phosphatidylinositol-3 kinase (PI3K) activation and epithelial–mesenchymal transition (EMT) (Park et al., 2017). Additionally, LPS is involved in the formation and alteration of local inflammation with elevated IL-1β and IL-6 through the activation of the NF-κB signaling pathway (Sun et al., 2018; Wang et al., 2024a). Beyond bacteria, viruses such as CMV, fungi such as *Aspergillus* and *Cladosporium*, and parasites such as *Dipylidium* have also been enriched in OC as detected by Patho-Chip sequencing with DNA (Banerjee et al., 2017). Fungi were detected using hematoxylin and eosin (HE) staining with antibodies against β-glucan, *Aspergillus*, CD45, CD68, and CD8, and fluorescence *in situ* hybridization (FISH) with probes against fungal 28*S* rRNA sequences (Narunsky-Haziza et al., 2022). CMV has been detected and shown to stimulate inflammation in borderline ovarian tumors (BOTs) through the potent 5-lipoxygenase, promoting BOT development via anti-apoptotic signaling pathways (Rahbar et al., 2021). However, there is a lack of direct tissue evidence for the presence of parasites. Given the high sensitivity of high-throughput sequencing techniques, sequencing of target genes for numerous microbiomes and subsequent bioinformatics matching may lead to a high false-positive rate and misinterpretation. In the future, more objective histological tests, such as immunohistochemical staining targeting specific flora, may be more helpful in identifying and characterizing the microbial composition in OC.

**Figure 2:**
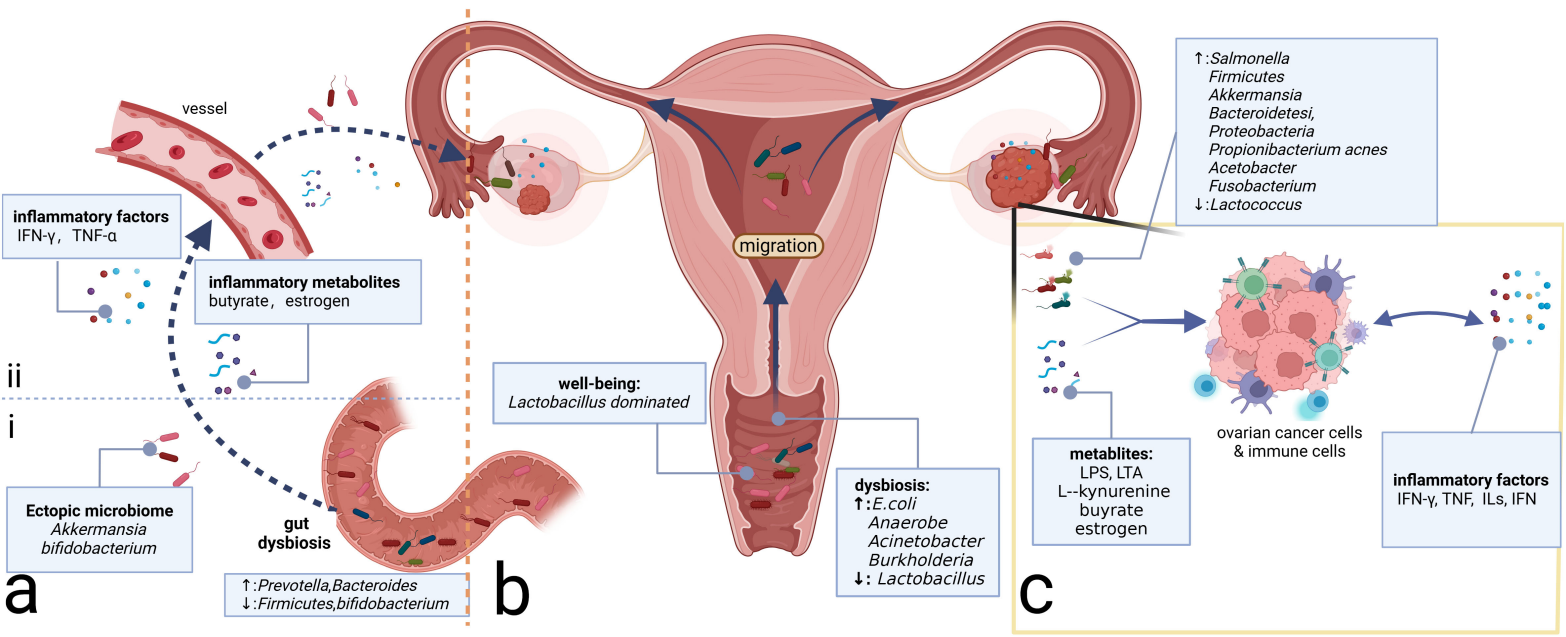
Microbiome and ovarian cancer. **(A)** Gut microbiome can affect the inflammatory microenvironment of ovarian cancer through direct and indirect effects. (i) Direct migration induced by intestinal epithelium-damaging via blood circulation. (ii) Intestinal microbiome-associated metabolites and secreted inflammatory factors alter the levels of the corresponding factors distantly. **(B)** Microbiomes vary from site to site in the reproductive tract, with microbial communities decreasing in number but increasing in diversity from the LRT to the URT. Migration of the microbiome can change the community in the ovary, which, in turn, alters the composition of the local inflammatory microenvironment. **(C)** Intratumor microbiome and metabolites such as LPS and LTA can impact the ovarian cancer cells and immune cells in the tumor microenvironment, prompting changes in the composition of inflammatory molecules such as TNF, ILs, and IFN, which, in turn, affects the immune cells’ constitution, inducing the alterations of the inflammatory microenvironment. LPS, lipopolysaccharide; LTA, lipoteichoic acid; TNF, tumor necrosis factor; ILs, interleukins; IFN, interferon; LRT, lower genital tract; URT, upper reproductive tract.

Similar to the disparity between cancerous and non-cancerous microbiomes, the microbiome in the metastatic niches often differs from that of the primary tumor site (Battaglia et al., 2024). In a pan-cancer analysis including OC, metastatic niches are characterized by higher microbiome diversity and elevated levels of transforming growth factor-β (TGF-β) and TNF-α, which induce inflammation and promote metastasis within the extracellular matrix (Battaglia et al., 2024). The mechanisms underlying microbial carcinogenesis in metastases may be analogous to those in primary focal niches. Bacteria-associated toxic metabolites, such as LPS on the surface of Gram-negative bacteria and lipoteichoic acid on the surface of Gram-positive bacteria, can induce the production of IL-6, TNF-α, and IFN, contributing to the formation of a pro-inflammatory microenvironment in metastatic niches (Sipos et al., 2021). Available studies suggest that the intratumor microbiome, whether in primary or metastatic niches, can promote OC progression. The potential mechanisms may involve microbiome-associated toxic metabolites and the accumulation of inflammatory factors, leading to alterations in the inflammatory immune microenvironment (**Figure 2C**). One possible mechanism is that the original microbiome at the metastatic niche may be tumor-suppressive, but the microbiome and their metabolites from the primary tumor niche could competitively inhibit the probiotics, potentially leading to the formation of a pro-tumoral pre-metastatic niche and promoting metastasis.

### 3.2 Gut microbiome and ovarian cancer

The gastrointestinal tract harbors the most diverse microbiome in the human body. The balanced composition of the gut microbiome is instrumental in maintaining immune and metabolic homeostasis, as well as resisting pathogens (Thursby and Juge, 2017). The gut microbiome of OC patients exhibits significant alterations compared to healthy individuals, characterized by a substantial decrease in bacterial diversity and an increase in opportunistic pathogens (Hu et al., 2023). Research has indicated that radical surgery and chemotherapy can modify the gut microbiome of OC patients (Tong et al., 2020). Platinum-based regimens, the standard scheme of OC chemotherapy, can reduce the abundance of *Firmicutes* in the gut (Tong et al., 2020), the bacterial group of which is elevated in OC patients (Hu et al., 2023). Antibiotic therapy can also modify the gut and vaginal microbiome, leading to a reduction in the abundance of *Prevotella* spp. in the fecal microbiota and potentially procrastinating the progression of high-grade serous OC (Chen et al., 2021). However, inappropriate antibiotic therapy that can disrupt and disorganize the intestinal microbiome may promote tumor growth and cisplatin resistance in OC (Chambers et al., 2022).

Alterations in the gut microbiome can shape the inflammatory environment of tumors through changes in immune cells and inflammatory mediators. Dysbiosis can stimulate the activation of tumor-associated macrophages (TAMs) and the accumulation of pro-inflammatory factors such as IL-6 and TNF-α, which promote OC progression by inducing EMT (Xu et al., 2019). Regarding specific genera, *Proteobacteria* such as *Prevotella* and *Bacteroides* are increased, while *Firmicutes* and *Bifidobacterium* are decreased (Hu et al., 2023). The increased presence of *Bacteroides* is essential for the production of butyrate in the intestine (Macfarlane and Macfarlane, 2003), which can regulate the function of CD8 T cells and induce the accumulation of IFN-γ and TNF-α in the TME (Zhu et al., 2023a). Pro-inflammatory factors such as TNF-α have been proven to promote EOC progression via the Hedgehog signaling pathway mediated by NF-κB signaling (Hu et al., 2023). In addition to inflammatory metabolites, alterations in the gut microbiome can also induce estrogenic perturbations and thus affect the OC microenvironment (Flores et al., 2012). Gut microbiomes such as *Bacteroides* and *Lactobacillus* secrete β-glucuronidase (GUS), which can regulate the circulating estrogen level (Wang et al., 2024b). Estrogen is suggested to be correlated with OC development by activating the estrogen receptor α (ERRα) and subsequent inflammatory pathways such as NF-κB/IL-6/STAT3 and PI3K/AKT signaling (Huang et al., 2022b). Beyond the alterations of microbial metabolites and inflammatory factors affecting the TME, the gut microbiome also interacts with the intratumor microbiome and may be beneficial in antitumor therapy. Zhu and colleagues demonstrate that *A. muciniphila* can migrate through the vessels and then proliferate in the primary tumor site to exert its antitumor efficacy by regulating the glutamine, purine, and pyrimidine metabolites (Zhu et al., 2023b). Additionally, the supplementation of *Akkermansia* through fecal transplantation in OC can elicit the activity of CD8+ T cells and induce the antitumor effect (Wang et al., 2022b). Similarly, oral *Bifidobacterium* supplementation can eventually accumulate in the TME and subsequently activate the STING (stimulator of interferon genes) and type I IFN signaling pathway, promoting the accumulation of IFN-β and the formation of the immune-responsive TME (Shi et al., 2020).

Above all, gut microbiome dysbiosis can activate inflammatory pathways through inflammatory metabolites and factors, thereby contributing to tumor promotion. Moreover, the gut microbiome can interact with the intratumor microbiome through direct migration, altering the TME microbiome composition (**Figure 2A**). However, gut microbiome dysbiosis is not yet definitively recognized as an etiology of OC. It may serve as a risk factor for OC potentially inducing the accumulation of harmful genetic mutations. Furthermore, the pelvic microenvironment characterized by abundant ascites in OC can influence the enteric environment through the ascites-mediated chronic inflammatory environment, facilitating a vicious cycle that promotes OC development.

### 3.3 Cavity microbiome and ovarian cancer

The ovaries, located deep within the abdominal cavity, are vital peritoneal organs with the surface covered by the peritoneum. Similar to the liver and pancreas, the ovaries communicate with the microbiome-rich sites such as the lower reproductive tract (LRT) (Li et al., 2023). Although the URT was once considered sterile, its non-sterile nature has also been confirmed by high-precision techniques (Verstraelen et al., 2016; Walther-António et al., 2016). This part will comb the microbiome present within these cavities and investigate its potential influence on the ovarian inflammatory microenvironment, with the goal of bridging the theoretical gaps in this field and identifying convenient detection methods and microbiome-based therapies.

#### 3.3.1 Peritoneal microbiome and ovarian cancer

Microbiomes are present on the surface of the ovaries. There are three potential origins: upward migration of the microbiome from the LRT, circulatory mediation by the circulatory system, and translocation from neighboring tissues such as the urinary and gut tracts (Qin et al., 2022; Sipos et al., 2021) (**Figure 2**). Ascites and peritoneal washings from OC patients exhibit decreased microbiome diversity. As for specific genera, *Salmonella*, *Bacteroidetes*, *Proteobacteria*, and *Akkermansia* are relatively enriched on the surface of the ovaries with tumor transformation (Miao et al., 2020; Qin et al., 2022). It has been confirmed that intraperitoneal injections of antibiotics targeting *Salmonella typhimurium* can reduce the abdominal spread of OC (Matsumoto et al., 2015). Meanwhile, engineered *S. typhimurium* has demonstrated antitumor potential targeting cancer cells (Tan et al., 2022). As the same peritoneal internal organs, the liver and pancreas also have channel-assisted communication with the microbiome-rich cavity. The peritoneal microbiome in hepatocellular carcinoma has been reported to activate the immune receptor TLR2 through metabolites such as deoxycholic acid and LTA inducing the formation of the immunosuppressive microenvironment (Loo et al., 2017; Ohtani and Hara, 2021). The intestinal origin *Malassezia globosa*, which is significantly enriched in pancreatic cancer tissue, can promote a complement cascade through its toxic products such as glycans of the fungal wall, thereby impairing the innate immune response and contributing to tumor progression (Aykut et al., 2019). These studies demonstrate that the microbial products can alter the inflammatory microenvironment through immune-based pathways. However, the mechanisms by which the peritoneal microbiome contributes to the OC are not fully understood. Considering the anatomical similarity between the ovary and the other peritoneal endodermal organs such as the liver and pancreas, as well as the fact that the microbiome can alter the TME through the aforementioned microbial metabolites, it is highly conceivable that the mechanisms of peritoneal microbiome carcinogenesis in OC may be inspired by those observed in the more extensively studied peritoneal endodermal organs.

#### 3.3.2 Microbiome of the reproductive tract

Similar to other mucosal sites in the body, the female reproductive tract harbors a specific microbiome community that is crucial for maintaining the health of the female reproductive system. In most women of reproductive age, the microbiota of the LRT (vagina and cervix) is predominantly composed of *Lactobacillus* spp (van de Wijgert et al., 2014), preserving the balance of the genital microenvironment and the health of the female reproductive tract. Conversely, the URT (uterus, fallopian tubes, and ovaries) was traditionally considered sterile. However, recent studies have confirmed the presence of microbiomes in the URT using advanced testing techniques (Verstraelen et al., 2016; Walther-António et al., 2016), which is generally characterized by lower microbial abundance but higher microbial diversity than the LRT (Chen et al., 2017) (**Figure 2B**). The reproductive tract microbiome can exert a distant influence on the TME of OC through proximal migration along the tract and alterations in inflammatory metabolites or factors.

It is now understood that microbiomes from the LRT can migrate to the URT and even the surface of the ovaries, contributing to the progression of OC (Li et al., 2023) (**Figure 2B**). The microbiome in the vagina of OC patients exhibits decreased *Lactobacillus* and increased *E. coli* compared to healthy women of the same age (Jacobson et al., 2021). The composition of the vaginal microbiome in patients with a good response to platinum-based chemotherapy differs from that in non-responders, which suggests that vaginal microbiome testing may be valuable in assessing the efficacy of platinum-based chemotherapy and predicting prognosis (Jacobson et al., 2021). Traditionally, *Lactobacillus* has been considered protective. However, studies have shown that *Lactobacillus iners* upregulates the glycolytic pathway in cervical cancer, producing large amounts of L-lactic acid under anaerobic conditions, which, in turn, induces chemo- and radiotherapy resistance (Johnston and Bullman, 2024) via the Warburg effect and glutamate and galactose metabolism pathways (Walther-António et al., 2016). This finding underscores the importance of distinguishing the *Lactobacillus* subtypes to better understand their underlying functions. Beyond *Lactobacillus*, vaginal microbiomes such as *Acinetobacter* and *Burkholderia* are enriched in OC and are correlated positively with L-kynurenine and negatively with L‐tyrosine (Li et al., 2023). However, further investigation is necessary to elucidate the precise mechanisms through which these metabolic alterations influence the TME in OC.

Considering the fallopian tubes' proximity to the ovaries and the fact that they share a similar germinal origin, the study of fallopian tube-associated microbiomes has been extensively emphasized. Previous research has shown that tubal ligation can reduce the risk of OC (Torre et al., 2018) and influence the distribution of microbiomes in the ovaries and fallopian tubes (Qin et al., 2022). A large prospective study has revealed that 60% of the top 20 most prevalent bacterial species in the fallopian tubes of OC patients originate primarily from the intestinal tract (Yu et al., 2024), suggesting potential communication between the reproductive and intestinal tracts. *Klebsiella* is most prevalent in non-plasmacytoid carcinoma, while *Anaerococcus* is the most prevalent in plasmacytoid carcinoma (Yu et al., 2024). Beyond bacteria, persistent chronic *Chlamydia trachomatis* infection can also contribute to OC development, which can induce CpG methylation to increase the cell stemness in the epithelial cells of the fallopian tubes (Kessler et al., 2019). Given the low microbial content of the URT and the susceptibility of sampling to be interfered by adjacent tissues, the accurate spectrum of the URT microbiome remains to be elucidated. Future research should prioritize extra attention and optimized specimen collection to control confounding factors.

## 4 Microbiome-associated inflammatory cells in ovarian cancer

The tumor inflammatory microenvironment comprises tumor cells, immune cells, stromal cells, and inflammatory factors (Mantovani et al., 2008). The infiltration of the specific immune cell types (Chen et al., 2023) and inflammatory factors is critical for the pro- or antitumor transformation. The exogenous pathogens and endogenous microbiome dysbiosis can also alter the levels of inflammatory factors and the function of immune cells (Battaglia et al., 2024; He et al., 2021; Huang et al., 2022a; Mantovani et al., 2008; Sipos et al., 2021; Wang et al., 2022b; Xu et al., 2019). Inflammatory-associated immune cells and their secreted inflammatory mediators are key contributors to the alterations in the inflammatory microenvironment. Thus, here we focused on the microbiome-associated immune cells exhibiting inflammatory changes.

### 4.1 The antigen-presenting immune cell alterations

**Figure 3:**
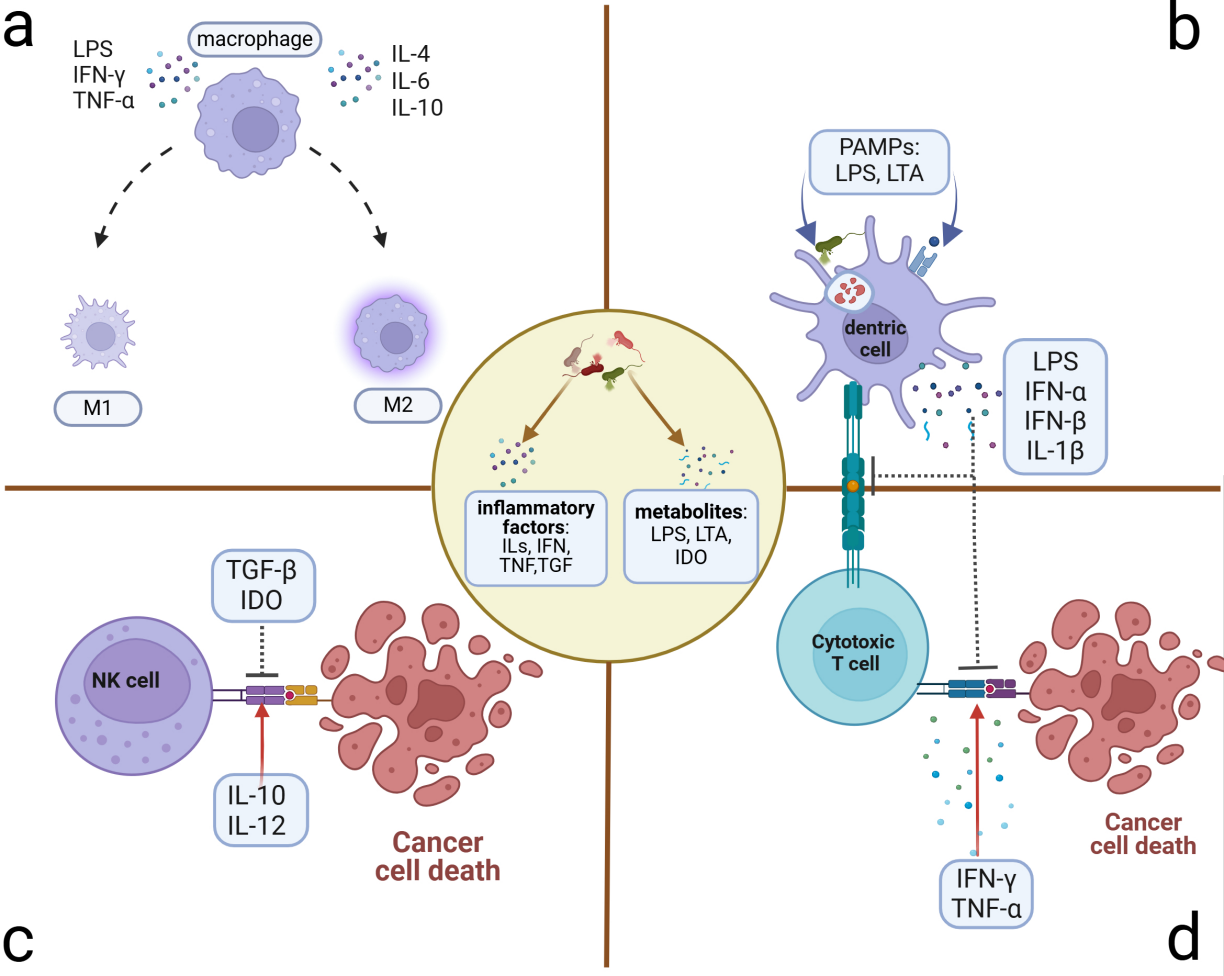
Microbiome-related inflammatory immune cell alterations. Microbiome and their stimulus on cancer or immune cells can exert many inflammatory metabolites and factors, which induce the alterations of immune cells in the TME. **(A)** Polarization of macrophages: M1 phenotype presenting with antitumor activity can be polarized by LPS, IFN-γ, and TNF-α. M2 phenotype presenting with pro-tumor activity can be polarized by ILs. **(B)** Suppression of antigen presentation by DC cells: Microbiome and the lysis products in and out of the DCs can activate the T cells through antigen presentation. However, some PAMPs can induce the accumulation of immune-suppressive factors, which can inhibit the antigen presentation of DCs and the killing activity of T cells. **(C)** Killing activity of NK cell alteration: Microbiome induces the transformation of dominant inflammatory factors in the microenvironment, some of which such as TGF-β and IDO can inhibit the killing activity to promote tumor progression. Others such as IL-10 and IL-12 can facilitate the killing activity. **(D)** Alteration of cytotoxic T cells that exert the killing activity through activation of APCs. Microbe-associated inflammatory factors can promote this process (e.g., IFN-γ and TNF-α) to hinder tumor progression or inhibit it (e.g., LPS, IFN, and IL-1β) to induce immune tolerance and tumor progression. DCs, dendritic cells; NK cells, natural killer cells; TGF, transforming growth factor; IDO, indoleamine 2,3-dioxygenase; APCs, antigen-presenting cells.

Traditional antigen-presenting cells (APCs), particularly TAMs and DCs within the TME, can exert the anti- or pro-tumor effects by sensing alterations in pathogen-associated molecular patterns (PAMPs), including the immunogenic bacterial composition, metabolites, and inflammatory factors generated by the microbiome (Fridlender et al., 2009; Hong et al., 2018; Lavin et al., 2015; Yan et al., 2023; Yuan et al., 2017). TAMs can be polarized into two main functional subtypes with opposing tumor effects influenced by the aforementioned molecules (**Figure 3A**). The bacterial constituent LPS (Locati et al., 2020; Wanderley et al., 2018) and the inflammatory factor IFN-γ can polarize TAMs toward the M1-type transformation (Liu et al., 2021) through the NF-κB and JAK2 pathways, respectively (Ji et al., 2019; Li and Verma, 2002; Wang et al., 2014). M1 can activate cytotoxic T lymphocytes through antigen presentation and secrete pro-inflammatory cytokines such as TNF-α to impede tumor development (Zhou et al., 2024). Additionally, they have been shown to promote IL-12-mediated Th1-type responses, further demonstrating their protective role in tumor development (Zhou et al., 2024). The IL family, such as IL-6, IL-4, and IL-10, can polarize TAMs toward the M2 type with the pro-tumor effect and diminished antigen-presenting ability (Biswas and Mantovani, 2010; Locati et al., 2020), which can decrease T-cell infiltration and promote the establishment of an immunosuppressive TME (Gottlieb et al., 2017). M2 also exhibits elevated glucose metabolism, which can enhance the production of mature histone B facilitating tumor metastasis and chemotherapy resistance (Shi et al., 2022). They can also induce angiogenesis to promote tumor advancement. As for the DCs, microbiome-related factors can inhibit their antigen-presenting function (**Figure 3B**). Lysophosphatidic acid (LPA) can inhibit the DC-mediated response to type I interferon (mainly containing IFN-α and IFN-β), promoting immunosuppression and tumor progression (Chae et al., 2022). The metabolite cyclic di-adenosine monophosphate from Live Gram-positive bacteria, a potent PAMP that facilitates classical autophagy by binding to APCs and subsequently activating the STING signaling pathway, assists in the accumulation of IL-1β in the TME (Moretti et al., 2017), which is significantly elevated in OC TME. Metabolism-associated insulin-like growth factor receptor (IGF1R), highly expressed in advanced OC cells and associated with viral invasion of mucosal tissues, can suppress antitumor immunity by inhibiting DC maturation (Griffiths et al., 2020; Somri-Gannam et al., 2020). Artificial immunogenic lipid-coated mesoporous silica nanoparticles (ILM) with PAMP properties can activate DCs and induce MHC presentation, which, in turn, promotes their uptake by myeloid cells and effectively increases the targeting accuracy of carried drugs (Noureddine et al., 2023). The injection of genetically engineered or specific tumor-targeted microbiomes into either the tumor tissue or the vein can delay tumor development. These microbiomes can induce macrophage migration into tumor tissues (Byrne et al., 2014) and activate the inhibited immune microenvironment through elevated IL-6, C-reactive protein (CRP), and vascular endothelial growth factor (VEGF) in systemic inflammation (Janku et al., 2021) or elevated metabolites such as L-arginine in the TME (Canale et al., 2021). The implementation of engineered or tumor-targeting microbiomes holds significant promise as a robust therapeutic supplement for chemotherapy and immunotherapy in recurrent OC patients by transforming the immune-inhibited microenvironment into an inflammatory immune-responsive microenvironment.

### 4.2 The cytocide immune cells alterations

As innate lymphocytes with cytocidal activity, natural killer (NK) cells respond rapidly by exerting direct killing (Crinier et al., 2020; Wu et al., 2020), which can be inhibited by microbiome-related factors (**Figure 3C**). Patients with advanced OC often present with ascites, which contain abundant inflammatory factors and cells in a hyperinflammatory state (Tonetti et al., 2021). In addition to ascites, the tumor niche is also composed of diverse microbiomes and elevated inflammatory factors such as TGF-β (Battaglia et al., 2024). Inflammatory molecules in the TME can affect NK cells’ cytocidal effects (Raja et al., 2022). Fraser et al. found that high levels of CA125 in the ascites and ovarian microenvironment can inhibit Fcγ receptor-mediated NK cell activation (Fraser et al., 2022). Metabolites such as indoleamine 2,3-dioxygenase (IDO) and inflammatory factors such as TGF-β can impair the killing ability of NK cells (Coyle et al., 2023; Hawke et al., 2020; Viel et al., 2016). Additionally, IL-2 and IL-15 can induce STAT5 (signal transducer and activator of transcription 5) signaling activation in NK cells, which, in turn, reduces the transcription and expression of the VEGF-A facilitating the killing activity of NK cells (Gotthardt et al., 2016). Conversely, IL-10 and IL-12 have been verified to reduce the phosphorylation of the STAT5 gene in NK cells, contributing to tumor progression by increasing the production of VEGF-A protein and subsequent vascular endothelial growth (Gotthardt et al., 2016). These findings suggest potential therapeutic avenues. The inhibitors of inflammatory molecules such as TGF-β can promote NK cells' cytocidal effects (Cully, 2020) and enhance the effectiveness of immune checkpoint inhibitors (ICIs) (Martin et al., 2020).

Regarding cytotoxic cells within adaptive immune cells, tumor-associated T cells can be broadly classified into cytotoxic T lymphocytes such as CD8+ T cells and helper T lymphocytes, the latter of which can be activated to secrete inflammatory factors such as IL-2, IL-16, and IFN-γ promoting the killing of toxic T lymphocytes (Nesbeth et al., 2010; Pinto et al., 2018). Alternatively, helper T lymphocytes can also secrete IL-4, IL-5, IL-6, and IL-10, inducing the formation of an immunosuppressive TME (Duraiswamy et al., 2021) (**Figure 3D**). Based on the infiltration scale of T cells, particularly those with cytotoxic function (Duraiswamy et al., 2021), TMEs can be categorized into three groups: the “hot” TME with abundant T-cell infiltration, the “excluded” TME with T-cell infiltration in the stroma zone but absent from the tumor site, and the “cold” TME with minimal or no T-cell infiltration (Chen and Mellman, 2017). The microbiome-associated immune cells contribute to TME transformation. APCs are essential for providing co-stimulatory signals to CD8+ T cells, facilitating their survival and the “hot” TME transformation (Duraiswamy et al., 2021). Previous research indicates that oral vancomycin can increase the CD8α+ DCs and IL-12 globally, optimizing the efficacy of adoptively transferred antitumor T-cell therapy (Uribe-Herranz et al., 2018). However, the specific targeted microbiome has not yet been identified. Therapy-induced changes in inflammatory factors in the circulatory system can also affect TME transformation. A preclinical study demonstrated that chemotherapy with a platinum-based regimen could induce the “hot” TME transformation and alleviate the immunosuppression by reducing circulating levels of IL-6 and enhancing the infiltration of TIL (2:1 mix of CD4+ and CD8+ T cells) infusion (Verdegaal et al., 2023). Meanwhile, LPS induces the elevation of IL-6 (Sipos et al., 2021), which may influence the tumor inflammatory microenvironment through a similar mechanism. In the M2-dominated microenvironment, the combination of atezolizumab and the novel ICI tiragolumab against TIGIT (T-cell immunoreceptor with Ig and ITIM domains) has been observed to increase IL-2 and TNF, thereby promoting T-cell infiltration and a “hot” TME transformation (Guan et al., 2024). The primary and metastatic niches, characterized by microbiome-induced elevated TNF (Battaglia et al., 2024; Huang et al., 2022a), may also alter the infiltration of T cells and consequently the according TME transformation. Microbiome-associated immune cells and the variations in inflammatory factors can trigger changes in T-cell infiltration and the TME transformation, which is a promising outlook for microbiome-associated inflammatory research.

## 5 Ovarian cancer-associated bacteria in therapy

A diverse range of microbiomes, including bacteria, fungi, and viruses, have been implicated in tumorigenesis and development. However, the majority of the current research on microbiome-related drugs focuses on bacteria. Anaerobic bacteria possess exceptional hypoxia-induced tumor-targeting properties and are amenable to be engineered. Consequently, most contemporary preclinical and clinical studies concentrate on anaerobic bacteria, among which *Escherichia coli*, *Bifidobacterium*, *Salmonella*, and *Bacillus* are the most commonly investigated and associated with OC. Bacteria with tumor necrosis properties, such as *Clostridium novyi* and *S. typhimurium* VNP20009 are also being explored (**Table 1**).

**Table 1:** OC-associated bacteria in therapy research for OC and other cancers.

Genus	Species	Effect	Modification/Usage	Cancer type	Phase	PMID
***Escherichia coli***	*E. coli* Nissle 1917	Targeted delivery Chemosensitization	Engineered to load 5-FU	Colon cancer	Preclinical study	36647536
	*E. coli*	Targeted delivery Chemosensitization	Engineered to load doxorubicin nanoparticles	Melanoma	Preclinical study	36563772
	*E. coli* Nissle 1917	Targeted delivery Cytolytic therapy	Engineered to load cytotoxic protein	Colon cancer	Preclinical study	33712706
	*E. coli* Nissle 1917	Targeted delivery M1 polarization	Engineered to load zoledronic acid	Breast cancer	Preclinical study	34171461
	*E. coli* Nissle 1917	DC maturation Cytotoxic T-cell activation	Bioreactor for intracellular immunity	Breast cancer	Preclinical study	34889048
	*E. coli* MG1655	M1 polarization Maturation of DCs	Engineered to load IFN-γ gene	Breast cancer	Preclinical study	38640931
***Bifidobacterium***	*Bifidobacterium infantis*	Radiosensitization	Given with its specific monoclonal antibody	Lung cancer	Preclinical study	33130941
	*Bifidobacterium infantis*	Targeted delivery Chemosensitization	Engineered to load cytosine deaminase gene	Lung cancer	Preclinical study	36945255
	*Bifidobacterium longum*	Targeted delivery Chemosensitization	Engineered to load 5-FU	Breast cancer	Preclinical study	16827806
***Bacillus***	*Bacillus* APS001F	Chemosensitization Prodrug conversion	Engineered to load cytosine deaminase gene	Solid tumors	Phase I/II study	NCT01562626
***Clostridium novyi***	*C. novyi*-NT	Inflammation Tumor necrosis	Intratumor injection	Leiomyosarcoma	Preclinical study	5122639
	*C. novyi*-NT	Systemic inflammation Tumor necrosis	Intratumor injection	Including OC	Phase I/II study	33046513
***Salmonella***	*S. typhimurium* VNP20009	Anoxic guidance Antitumor activity	VNP20009 mutant	Melanoma	Phase I study	16000580
	*S. typhimurium*	Better targeted Antitumor activity	Engineered to be tryptophan auxotrophic	Breast cancer	Preclinical study	35694447
	*S. typhimurium* *ΔppGpp*	Increase the tumor cell killing efficacy	Combined injection with IFN-γ	Colon cancer	Preclinical study	36246355
	*S. typhimurium* VNP20009	Increase the tumor cell killing efficacy	Macrophage–microbe encapsulation	Breast cancer, melanoma colon cancer	Preclinical study	39023382
	*S. typhimurium* VNP20009	Increase the tumor cell killing efficacy	Engineered shCCDC25 into VNP-shCCDC25	Lung metastasis cancer	Preclinical study	38369519

Bacteria can exert antitumor activity through three primary mechanisms: (1) engineering them to deliver chemotherapeutic drugs for enhanced efficacy, (2) inducing the transformation of the TME into an immune-responsive state, and (3) facilitating tumor necrosis through intratumor injection. In terms of cancer type, studies have focused on breast, colorectal, and lung cancers, all of which have been shown to be associated with bacteria prior to OC. Chemotherapeutic drugs such as doxorubicin, 5-FU, and precursor enzymes like cytosine deaminase can be targeted to the tumor niche by *E. coli* and *Bifidobacterium*, thereby increasing the effectiveness of chemotherapy treatment (Chen et al., 2023; Sasaki et al., 2006; Xie et al., 2021) (NCT01562626). When engineered with inflammatory factors or their genes, *E. coli* can promote the transformation of immune cells such as DCs and TAMs, fostering the formation of an immune-responsive TME (Xie et al., 2021; Yang et al., 2024; Yao et al., 2022). *C. novyi-NT* and *S. typhimurium* VNP20009 are capable of inducing tumor necrosis through intratumoral or intravenous injection (Janku et al., 2021; Roberts et al., 2014; Thamm et al., 2005). Our bodies are naturally resistant to exogenous bacteria, which can limit the effectiveness of therapeutic bacteria. To address this, researchers are exploring solutions such as injecting IFN-γ to reduce neutrophil killing (Xu et al., 2022). Additionally, coupling therapeutic bacteria with macrophages to reduce immunogenicity and transferring specific gene fragments into *S. typhimurium* VNP20009 to decrease the number of extracellular neutrophil traps may enable more therapeutic bacteria to enter the tumor niche (Liu et al., 2024; Wu et al., 2024).

Overall, bacteria-specific therapies for OC are currently less explored, and most research in other cancer types are also in the preclinical stage. Nevertheless, results from animal models demonstrate significant antitumor efficacy, and the utilization of specific bacteria to target the tumor niche for precise treatment holds promise for future clinical applications.

## 6 Conclusion

The phenomenon of inflammation at the tumor site has emerged as a prominent area of research interest. Similarly, research on the tumor-associated microbiome is also experiencing substantial growth. The presence of diverse dominant microbiomes in different parts of the female genital tract and the communication between the upper and lower genital tract offer a theoretical basis for future prediction of OC prognosis and early diagnosis through vaginal specimen analysis. However, further investigation is needed to elucidate the specific microbiome spectrum and associated biochemical indexes. Currently, most microbiome research related to OC is conducted in a general manner, and the specific spectrum of highly pathogenic microbiomes remains unclear. Consequently, research on the underlying mechanisms is still insufficient. The chemotaxis of inflammatory mediators and the activation of inflammatory pathways induced by similar microbiome disorders in more extensively studied cancers such as liver and pancreas cancers may provide valuable insights into the basic mechanisms of carcinogenesis in OC. Meanwhile, immune cells, the primary defenders against microbial infections, can undergo phenotypic transformations mediated by inflammatory factors and microbiome virulence metabolites within the TME, offering future insights into the potential mechanisms of microbiome-associated OC. In the future, studies focusing on the detailed mechanisms of microbiome-induced inflammatory and immune alterations may facilitate the deeper understanding of microbial tumor-promoting effects and assist us in the identification of probiotics and the optimization of microbial-associated immunotherapies. Furthermore, numerous investigations have explored the potential of bacteriotherapy to exert a direct killing effect or enhance the precision of chemotherapy or immunotherapy. This involves engineering bacteria to express targeted molecules, thereby improving the efficacy of these treatments. Interestingly, some of the same bacteria used for targeted therapy have also been identified as potential factors in OC, suggesting avenues for further research into this refractory disease.

## References

[B1] Ansaldo E. Slayden L. C. Ching K. L. Koch M. A. Wolf N. K. Plichta D. R.. (2019). Akkermansia muciniphila induces intestinal adaptive immune responses during homeostasis. Science 364, 1179–1184. doi: 10.1126/science.aaw7479 31221858 PMC6645389

[B2] Atarashi K. Tanoue T. Shima T. Imaoka A. Kuwahara T. Momose Y.. (2011). Induction of colonic regulatory T cells by indigenous Clostridium species. Science 331, 337–341. doi: 10.1126/science.1198469 21205640 PMC3969237

[B3] Aykut B. Pushalkar S. Chen R. Li Q. Abengozar R. Kim J. I.. (2019). The fungal mycobiome promotes pancreatic oncogenesis via activation of MBL. Nature 574, 264–267. doi: 10.1038/s41586-019-1608-2 31578522 PMC6858566

[B4] Banerjee S. Tian T. Wei Z. Shih N. Feldman M. D. Alwine J. C.. (2017). The ovarian cancer oncobiome. Oncotarget 8, 36225–36245. doi: 10.18632/oncotarget.16717 28410234 PMC5482651

[B5] Battaglia T. W. Mimpen I. L. Traets J. J. H. van Hoeck A. Zeverijn L. J. Geurts B. S.. (2024). A pan-cancer analysis of the microbiome in metastatic cancer. Cell 187, 2324–2335.e19. doi: 10.1016/j.cell.2024.03.021 38599211

[B6] Biswas S. K. Mantovani A. (2010). Macrophage plasticity and interaction with lymphocyte subsets: cancer as a paradigm. Nat. Immunol. 11, 889–896. doi: 10.1038/ni.1937 20856220

[B7] Bray F. Laversanne M. Sung H. Ferlay J. Siegel R. L. Soerjomataram I.. (2024). Global cancer statistics 2022: GLOBOCAN estimates of incidence and mortality worldwide for 36 cancers in 185 countries. CA Cancer J. Clin. 74, 229–263. doi: 10.3322/caac.21834 38572751

[B8] Buys S. S. Partridge E. Black A. Johnson C. C. Lamerato L. Isaacs C.. (2011). Effect of screening on ovarian cancer mortality: the Prostate, Lung, Colorectal and Ovarian (PLCO) Cancer Screening Randomized Controlled Trial. Jama 305, 2295–2303. doi: 10.1001/jama.2011.766 21642681

[B9] Byrne W. L. Murphy C. T. Cronin M. Wirth T. Tangney M. (2014). Bacterial-mediated DNA delivery to tumour associated phagocytic cells. J. Control Release 196, 384–393. doi: 10.1016/j.jconrel.2014.10.030 25466954

[B10] Canale F. P. Basso C. Antonini G. Perotti M. Li N. Sokolovska A.. (2021). Metabolic modulation of tumours with engineered bacteria for immunotherapy. Nature 598, 662–666. doi: 10.1038/s41586-021-04003-2 34616044

[B11] Chae C. S. Sandoval T. A. Hwang S. M. Park E. S. Giovanelli P. Awasthi D.. (2022). Tumor-derived lysophosphatidic acid blunts protective type I interferon responses in ovarian cancer. Cancer Discovery 12, 1904–1921. doi: 10.1158/2159-8290.CD-21-1181 35552618 PMC9357054

[B12] Chambers L. M. Esakov Rhoades E. L. Bharti R. Braley C. Tewari S. Trestan L.. (2022). Disruption of the gut microbiota confers cisplatin resistance in epithelial ovarian cancer. Cancer Res. 82, 4654–4669. doi: 10.1158/0008-5472.CAN-22-0455 36206317 PMC9772178

[B13] Chaplin D. D. (2010). Overview of the immune response. J. Allergy Clin. Immunol. 125, S3–23. doi: 10.1016/j.jaci.2009.12.980 20176265 PMC2923430

[B14] Chen C. Song X. Wei W. Zhong H. Dai J. Lan Z.. (2017). The microbiota continuum along the female reproductive tract and its relation to uterine-related diseases. Nat. Commun. 8, 875. doi: 10.1038/s41467-017-00901-0 29042534 PMC5645390

[B15] Chen D. S. Mellman I. (2017). Elements of cancer immunity and the cancer-immune set point. Nature 541, 321–330. doi: 10.1038/nature21349 28102259

[B16] Chen H. Lei P. Ji H. Yang Q. Peng B. Ma J.. (2023). Advances in Escherichia coli Nissle 1917 as a customizable drug delivery system for disease treatment and diagnosis strategies. Mater Today Bio 18, 100543. doi: 10.1016/j.mtbio.2023.100543 PMC9840185 36647536

[B17] Chen L. Zhai Y. Wang Y. Fearon E. R. Núñez G. Inohara N.. (2021). Altering the microbiome inhibits tumorigenesis in a mouse model of oviductal high-grade serous carcinoma. Cancer Res. 81, 3309–3318. doi: 10.1158/0008-5472.CAN-21-0106 33863776 PMC8260454

[B18] Coyle K. M. Hawke L. G. Ormiston M. L. (2023). Addressing natural killer cell dysfunction and plasticity in cell-based cancer therapeutics. Cancers (Basel) 15, 1743. doi: 10.3390/cancers15061743 36980629 PMC10046032

[B19] Crinier A. Narni-Mancinelli E. Ugolini S. Vivier E. (2020). SnapShot: natural killer cells. Cell 180, 1280–1280.e1. doi: 10.1016/j.cell.2020.02.029 32200803

[B20] Cully M. (2020). TGFβ1-specific antibody spurs anti-tumour immunity. Nat. Rev. Drug Discovery 19, 310. doi: 10.1038/d41573-020-00058-4 32246134

[B21] de Vos W. M. Tilg H. Van Hul M. Cani P. D. (2022). Gut microbiome and health: mechanistic insights. Gut 71, 1020–1032. doi: 10.1136/gutjnl-2021-326789 35105664 PMC8995832

[B22] Duraiswamy J. Turrini R. Minasyan A. Barras D. Crespo I. Grimm A. J.. (2021). Myeloid antigen-presenting cell niches sustain antitumor T cells and license PD-1 blockade via CD28 costimulation. Cancer Cell 39, 1623–1642.e20. doi: 10.1016/j.ccell.2021.10.008 34739845 PMC8861565

[B23] Dvorak H. F. (2015). Tumors: wounds that do not heal-redux. Cancer Immunol. Res. 3, 1–11. doi: 10.1158/2326-6066.CIR-14-0209 25568067 PMC4288010

[B24] Elinav E. Nowarski R. Thaiss C. A. Hu B. Jin C. Flavell R. A. (2013). Inflammation-induced cancer: crosstalk between tumours, immune cells and microorganisms. Nat. Rev. Cancer 13, 759–771. doi: 10.1038/nrc3611 24154716

[B25] Fathalla M. F. (1971). Incessant ovulation–a factor in ovarian neoplasia? Lancet 2, 163. doi: 10.1016/S0140-6736(71)92335-X 4104488

[B26] Fathalla M. F. (2016). Non-hormonal interruption of incessant ovulation as a potential approach for ovarian cancer prevention. Int. J. Gynaecol Obstet 132, 356–358. doi: 10.1016/j.ijgo.2015.11.006 26876699

[B27] Flores R. Shi J. Fuhrman B. Xu X. Veenstra T. D. Gail M. H.. (2012). Fecal microbial determinants of fecal and systemic estrogens and estrogen metabolites: a cross-sectional study. J. Transl. Med. 10, 253. doi: 10.1186/1479-5876-10-253 23259758 PMC3552825

[B28] Fraser C. C. Jia B. Hu G. Al Johani L. I. Fritz-Klaus R. Ham J. D.. (2022). Ovarian cancer ascites inhibits transcriptional activation of NK cells partly through CA125. J. Immunol. 208, 2227–2238. doi: 10.4049/jimmunol.2001095 35396222 PMC10852100

[B29] Fridlender Z. G. Sun J. Kim S. Kapoor V. Cheng G. Ling L.. (2009). Polarization of tumor-associated neutrophil phenotype by TGF-beta: "N1" versus "N2" TAN. Cancer Cell 16, 183–194. doi: 10.1016/j.ccr.2009.06.017 19732719 PMC2754404

[B30] Fu Y. Lyu J. Wang S. (2023). The role of intestinal microbes on intestinal barrier function and host immunity from a metabolite perspective. Front. Immunol. 14, 1277102. doi: 10.3389/fimmu.2023.1277102 37876938 PMC10591221

[B31] González-Martín A. Harter P. Leary A. Lorusso D. Miller R. E. Pothuri B.. (2023). Newly diagnosed and relapsed epithelial ovarian cancer: ESMO Clinical Practice Guideline for diagnosis, treatment and follow-up. Ann. Oncol. 34, 833–848. doi: 10.1016/j.annonc.2023.07.011 37597580

[B32] Gotthardt D. Putz E. M. Grundschober E. Prchal-Murphy M. Straka E. Kudweis P.. (2016). STAT5 is a key regulator in NK cells and acts as a molecular switch from tumor surveillance to tumor promotion. Cancer Discovery 6, 414–429. doi: 10.1158/2159-8290.CD-15-0732 26873347

[B33] Gottlieb C. E. Mills A. M. Cross J. V. Ring K. L. (2017). Tumor-associated macrophage expression of PD-L1 in implants of high grade serous ovarian carcinoma: A comparison of matched primary and metastatic tumors. Gynecol Oncol. 144, 607–612. doi: 10.1016/j.ygyno.2016.12.021 28065619

[B34] Greten F. R. Grivennikov S. I. (2019). Inflammation and cancer: triggers, mechanisms, and consequences. Immunity 51, 27–41. doi: 10.1016/j.immuni.2019.06.025 31315034 PMC6831096

[B35] Griffiths C. D. Bilawchuk L. M. McDonough J. E. Jamieson K. C. Elawar F. Cen Y.. (2020). IGF1R is an entry receptor for respiratory syncytial virus. Nature 583, 615–619. doi: 10.1038/s41586-020-2369-7 32494007

[B36] Guan X. Hu R. Choi Y. Srivats S. Nabet B. Y. Silva J.. (2024). Anti-TIGIT antibody improves PD-L1 blockade through myeloid and T(reg) cells. Nature 627, 646–655. doi: 10.1038/s41586-024-07121-9 38418879 PMC11139643

[B37] Hashimoto M. Kamphorst A. O. Im S. J. Kissick H. T. Pillai R. N. Ramalingam S. S.. (2018). CD8 T cell exhaustion in chronic infection and cancer: opportunities for interventions. Annu. Rev. Med. 69, 301–318. doi: 10.1146/annurev-med-012017-043208 29414259

[B38] Hawke L. G. Mitchell B. Z. Ormiston M. L. (2020). TGF-β and IL-15 synergize through MAPK pathways to drive the conversion of human NK cells to an innate lymphoid cell 1-like phenotype. J. Immunol. 204, 3171–3181. doi: 10.4049/jimmunol.1900866 32332109

[B39] He Y. Fu L. Li Y. Wang W. Gong M. Zhang J.. (2021). Gut microbial metabolites facilitate anticancer therapy efficacy by modulating cytotoxic CD8(+) T cell immunity. Cell Metab. 33, 988–1000.e7. doi: 10.1016/j.cmet.2021.03.002 33761313

[B40] Hong L. Wang S. Li W. Wu D. Chen W. (2018). Tumor-associated macrophages promote the metastasis of ovarian carcinoma cells by enhancing CXCL16/CXCR6 expression. Pathol. Res. Pract. 214, 1345–1351. doi: 10.1016/j.prp.2018.07.009 30049511

[B41] Hu X. Xu X. Zeng X. Jin R. Wang S. Jiang H.. (2023). Gut microbiota dysbiosis promotes the development of epithelial ovarian cancer via regulating Hedgehog signaling pathway. Gut Microbes 15, 2221093. doi: 10.1080/19490976.2023.2221093 37282604 PMC10249449

[B42] Huang W. Chen L. Sun P. (2022b). ERRα expression in ovarian cancer and promotes ovarian cancer cells migration in *vitro* . Arch. Gynecol Obstet 305, 1525–1534. doi: 10.1007/s00404-021-06323-0 34797420

[B43] Huang Q. Wei X. Li W. Ma Y. Chen G. Zhao L.. (2022a). Endogenous propionibacterium acnes promotes ovarian cancer progression via regulating hedgehog signalling pathway. Cancers (Basel) 14, 5178. doi: 10.3390/cancers14215178 36358596 PMC9658903

[B44] Jacobson D. Moore K. Gunderson C. Rowland M. Austin R. Honap T. P.. (2021). Shifts in gut and vaginal microbiomes are associated with cancer recurrence time in women with ovarian cancer. PeerJ 9, e11574. doi: 10.7717/peerj.11574 34178459 PMC8214851

[B45] Janku F. Zhang H. H. Pezeshki A. Goel S. Murthy R. Wang-Gillam A.. (2021). Intratumoral injection of clostridium novyi-NT spores in patients with treatment-refractory advanced solid tumors. Clin. Cancer Res. 27, 96–106. doi: 10.1158/1078-0432.CCR-20-2065 33046513

[B46] Ji L. Zhao X. Zhang B. Kang L. Song W. Zhao B.. (2019). Slc6a8-mediated creatine uptake and accumulation reprogram macrophage polarization via regulating cytokine responses. Immunity 51, 272–284.e7. doi: 10.1016/j.immuni.2019.06.007 31399282

[B47] Johnston C. D. Bullman S. (2024). Bacteria-derived L-lactate fuels cervical cancer chemoradiotherapy resistance. Trends Cancer 10, 97–99. doi: 10.1016/j.trecan.2024.01.001 38242824

[B48] Kashani B. Zandi Z. Bashash D. Zaghal A. Momeny M. Poursani E. M.. (2020). Small molecule inhibitor of TLR4 inhibits ovarian cancer cell proliferation: new insight into the anticancer effect of TAK-242 (Resatorvid). Cancer Chemother. Pharmacol. 85, 47–59. doi: 10.1007/s00280-019-03988-y 31786654

[B49] Kawahara N. Yamanaka S. Nishikawa K. Matsuoka M. Maehana T. Kawaguchi R.. (2024). Endogenous microbacteria can contribute to ovarian carcinogenesis by reducing iron concentration in cysts: A pilot study. Microorganisms 12, 538. doi: 10.3390/microorganisms12030538 38543589 PMC10975009

[B50] Kessler M. Hoffmann K. Fritsche K. Brinkmann V. Mollenkopf H. J. Thieck O.. (2019). Chronic Chlamydia infection in human organoids increases stemness and promotes age-dependent CpG methylation. Nat. Commun. 10, 1194. doi: 10.1038/s41467-019-09144-7 30886143 PMC6423033

[B51] Kostic A. D. Chun E. Robertson L. Glickman J. N. Gallini C. A. Michaud M.. (2013). Fusobacterium nucleatum potentiates intestinal tumorigenesis and modulates the tumor-immune microenvironment. Cell Host Microbe 14, 207–215. doi: 10.1016/j.chom.2013.07.007 23954159 PMC3772512

[B52] Lavin Y. Mortha A. Rahman A. Merad M. (2015). Regulation of macrophage development and function in peripheral tissues. Nat. Rev. Immunol. 15, 731–744. doi: 10.1038/nri3920 26603899 PMC4706379

[B53] Li C. Feng Y. Yang C. Wang D. Zhang D. Luo X.. (2023). Association between vaginal microbiota and the progression of ovarian cancer. J. Med. Virol. 95, e28898. doi: 10.1002/jmv.28898 37409619

[B54] Li Q. Verma I. M. (2002). NF-kappaB regulation in the immune system. Nat. Rev. Immunol. 2, 725–734. doi: 10.1038/nri910 12360211

[B55] Lin H. W. Tu Y. Y. Lin S. Y. Su W. J. Lin W. L. Lin W. Z.. (2011). Risk of ovarian cancer in women with pelvic inflammatory disease: a population-based study. Lancet Oncol. 12, 900–904. doi: 10.1016/S1470-2045(11)70165-6 21835693

[B56] Liu L. N. Chen C. Xin W. J. Li Q. Han C. Hua Z. C. (2024). The oncolytic bacteria-mediated delivery system of CCDC25 nucleic acid drug inhibits neutrophil extracellular traps induced tumor metastasis. J. Nanobiotechnology 22, 69. doi: 10.1186/s12951-024-02335-5 38369519 PMC10875894

[B57] Liu Y. Fu K. Wier E. M. Lei Y. Hodgson A. Xu D.. (2022). Bacterial genotoxin accelerates transient infection-driven murine colon tumorigenesis. Cancer Discovery 12, 236–249. doi: 10.1158/2159-8290.CD-21-0912 34479870 PMC8758537

[B58] Liu J. Geng X. Hou J. Wu G. (2021). New insights into M1/M2 macrophages: key modulators in cancer progression. Cancer Cell Int. 21, 389. doi: 10.1186/s12935-021-02089-2 34289846 PMC8296555

[B59] Liu Y. Metzinger M. N. Lewellen K. A. Cripps S. N. Carey K. D. Harper E. I.. (2015). Obesity contributes to ovarian cancer metastatic success through increased lipogenesis, enhanced vascularity, and decreased infiltration of M1 macrophages. Cancer Res. 75, 5046–5057. doi: 10.1158/0008-5472.CAN-15-0706 26573796 PMC4668203

[B60] Locati M. Curtale G. Mantovani A. (2020). Diversity, mechanisms, and significance of macrophage plasticity. Annu. Rev. Pathol. 15, 123–147. doi: 10.1146/annurev-pathmechdis-012418-012718 31530089 PMC7176483

[B61] Loo T. M. Kamachi F. Watanabe Y. Yoshimoto S. Kanda H. Arai Y.. (2017). Gut microbiota promotes obesity-associated liver cancer through PGE(2)-mediated suppression of antitumor immunity. Cancer Discovery 7, 522–538. doi: 10.1158/2159-8290.CD-16-0932 28202625

[B62] Macciò A. Madeddu C. (2012). Inflammation and ovarian cancer. Cytokine 58, 133–147. doi: 10.1016/j.cyto.2012.01.015 22349527

[B63] Macfarlane S. Macfarlane G. T. (2003). Regulation of short-chain fatty acid production. Proc. Nutr. Soc. 62, 67–72. doi: 10.1079/PNS2002207 12740060

[B64] Malesza I. J. Malesza M. Walkowiak J. Mussin N. Walkowiak D. Aringazina R.. (2021). High-fat, western-style diet, systemic inflammation, and gut microbiota: A narrative review. Cells 10, 3164. doi: 10.3390/cells10113164 34831387 PMC8619527

[B65] Mantovani A. Allavena P. Sica A. Balkwill F. (2008). Cancer-related inflammation. Nature 454, 436–444. doi: 10.1038/nature07205 18650914

[B66] Martin C. J. Datta A. Littlefield C. Kalra A. Chapron C. Wawersik S.. (2020). Selective inhibition of TGFβ1 activation overcomes primary resistance to checkpoint blockade therapy by altering tumor immune landscape. Sci. Transl. Med. 12, eaay8456. doi: 10.1126/scitranslmed.aay8456 32213632

[B67] Matsumoto Y. Miwa S. Zhang Y. Zhao M. Yano S. Uehara F.. (2015). Intraperitoneal administration of tumor-targeting Salmonella typhimurium A1-R inhibits disseminated human ovarian cancer and extends survival in nude mice. Oncotarget 6, 11369–11377. doi: 10.18632/oncotarget.v6i13 25957417 PMC4484462

[B68] Medzhitov R. (2008). Origin and physiological roles of inflammation. Nature 454, 428–435. doi: 10.1038/nature07201 18650913

[B69] Menon U. Gentry-Maharaj A. Burnell M. Singh N. Ryan A. Karpinskyj C.. (2021). Ovarian cancer population screening and mortality after long-term follow-up in the UK Collaborative Trial of Ovarian Cancer Screening (UKCTOCS): a randomised controlled trial. Lancet 397, 2182–2193. doi: 10.1016/S0140-6736(21)00731-5 33991479 PMC8192829

[B70] Menon U. Ryan A. Kalsi J. Gentry-Maharaj A. Dawnay A. Habib M.. (2015). Risk algorithm using serial biomarker measurements doubles the number of screen-detected cancers compared with a single-threshold rule in the United Kingdom collaborative trial of ovarian cancer screening. J. Clin. Oncol. 33, 2062–2071. doi: 10.1200/JCO.2014.59.4945 25964255 PMC4463475

[B71] Miao R. Badger T. C. Groesch K. Diaz-Sylvester P. L. Wilson T. Ghareeb A.. (2020). Assessment of peritoneal microbial features and tumor marker levels as potential diagnostic tools for ovarian cancer. PloS One 15, e0227707. doi: 10.1371/journal.pone.0227707 31917801 PMC6952086

[B72] Moretti J. Roy S. Bozec D. Martinez J. Chapman J. R. Ueberheide B.. (2017). STING senses microbial viability to orchestrate stress-mediated autophagy of the endoplasmic reticulum. Cell 171, 809–823.e13. doi: 10.1016/j.cell.2017.09.034 29056340 PMC5811766

[B73] Narunsky-Haziza L. Sepich-Poore G. D. Livyatan I. Asraf O. Martino C. Nejman D.. (2022). Pan-cancer analyses reveal cancer-type-specific fungal ecologies and bacteriome interactions. Cell 185, 3789–3806.e17. doi: 10.1016/j.cell.2022.09.005 36179670 PMC9567272

[B74] Nejman D. Livyatan I. Fuks G. Gavert N. Zwang Y. Geller L. T.. (2020). The human tumor microbiome is composed of tumor type-specific intracellular bacteria. Science 368, 973–980. doi: 10.1126/science.aay9189 32467386 PMC7757858

[B75] Nené N. R. Reisel D. Leimbach A. Franchi D. Jones A. Evans I.. (2019). Association between the cervicovaginal microbiome, BRCA1 mutation status, and risk of ovarian cancer: a case-control study. Lancet Oncol. 20, 1171–1182. doi: 10.1016/S1470-2045(19)30340-7 31300207

[B76] Nesbeth Y. C. Martinez D. G. Toraya S. Scarlett U. K. Cubillos-Ruiz J. R. Rutkowski M. R.. (2010). CD4+ T cells elicit host immune responses to MHC class II-negative ovarian cancer through CCL5 secretion and CD40-mediated licensing of dendritic cells. J. Immunol. 184, 5654–5662. doi: 10.4049/jimmunol.0903247 20400704 PMC2874073

[B77] Ness R. B. Grisso J. A. Cottreau C. Klapper J. Vergona R. Wheeler J. E.. (2000). Factors related to inflammation of the ovarian epithelium and risk of ovarian cancer. Epidemiology 11, 111–117. doi: 10.1097/00001648-200003000-00006 11021606

[B78] Niu T. Zhou F. (2023). Inflammation and tumor microenvironment. Zhong Nan Da Xue Xue Bao Yi Xue Ban 48, 1899–1913. doi: 10.11817/j.issn.1672-7347.2023.230231 38448384 PMC10930746

[B79] Noureddine A. Marwedel B. Tang L. Medina L. Y. Serda R. E. (2023). Specific tumor localization of immunogenic lipid-coated mesoporous silica nanoparticles following intraperitoneal administration in a mouse model of serous epithelial ovarian cancer. Cancers (Basel) 15, 4626. doi: 10.3390/cancers15184626 37760595 PMC10526288

[B80] Ohtani N. Hara E. (2021). Gut-liver axis-mediated mechanism of liver cancer: A special focus on the role of gut microbiota. Cancer Sci. 112, 4433–4443. doi: 10.1111/cas.v112.11 34533882 PMC8586687

[B81] Paavonen J. Turzanski Fortner R. Lehtinen M. Idahl A. (2021). Chlamydia trachomatis, pelvic inflammatory disease, and epithelial ovarian cancer. J. Infect. Dis. 224, S121–s127. doi: 10.1093/infdis/jiab017 34396414

[B82] Park G. B. Chung Y. H. Kim D. (2017). Induction of galectin-1 by TLR-dependent PI3K activation enhances epithelial-mesenchymal transition of metastatic ovarian cancer cells. Oncol. Rep. 37, 3137–3145. doi: 10.3892/or.2017.5533 28350104

[B83] Parkin D. M. Hämmerl L. Ferlay J. Kantelhardt E. J. (2020). Cancer in Africa 2018: The role of infections. Int. J. Cancer 146, 2089–2103. doi: 10.1002/ijc.v146.8 31254479

[B84] Peña O. A. Martin P. (2024). Cellular and molecular mechanisms of skin wound healing. Nat. Rev. Mol. Cell Biol 25, 599–616. doi: 10.1038/s41580-024-00715-1 38528155

[B85] Pinto M. P. Balmaceda C. Bravo M. L. Kato S. Villarroel A. Owen G. I.. (2018). Patient inflammatory status and CD4+/CD8+ intraepithelial tumor lymphocyte infiltration are predictors of outcomes in high-grade serous ovarian cancer. Gynecol Oncol. 151, 10–17. doi: 10.1016/j.ygyno.2018.07.025 30078505

[B86] Qin X. Zhou J. Wang Z. Feng C. Fan J. Huang J.. (2022). Metagenomic analysis of the microbiome of the upper reproductive tract: combating ovarian cancer through predictive, preventive, and personalized medicine. Epma J. 13, 487–498. doi: 10.1007/s13167-022-00286-1 35762010 PMC9219379

[B87] Rahbar A. Pantalone M. R. Religa P. Rådestad A. F. Söderberg-Naucler C. (2021). Evidence of human cytomegalovirus infection and expression of 5-lipoxygenase in borderline ovarian tumors. J. Med. Virol. 93, 4023–4027. doi: 10.1002/jmv.26664 33174621

[B88] Raja R. Wu C. Bassoy E. Y. Rubino T. E. Jr. Utagawa E. C. Magtibay P. M.. (2022). PP4 inhibition sensitizes ovarian cancer to NK cell-mediated cytotoxicity via STAT1 activation and inflammatory signaling. J. Immunother. Cancer 10, e005026. doi: 10.1136/jitc-2022-005026 36564125 PMC9791393

[B89] Roberts N. J. Zhang L. Janku F. Collins A. Bai R. Y. Staedtke V.. (2014). Intratumoral injection of Clostridium novyi-NT spores induces antitumor responses. Sci. Transl. Med. 6, 249ra111. doi: 10.1126/scitranslmed.3008982 PMC4399712 25122639

[B90] Sasaki T. Fujimori M. Hamaji Y. Hama Y. Ito K. Amano J.. (2006). Genetically engineered Bifidobacterium longum for tumor-targeting enzyme-prodrug therapy of autochthonous mammary tumors in rats. Cancer Sci. 97, 649–657. doi: 10.1111/j.1349-7006.2006.00221.x 16827806 PMC11159642

[B91] Schäfer M. Werner S. (2008). Cancer as an overhealing wound: an old hypothesis revisited. Nat. Rev. Mol. Cell Biol. 9, 628–638. doi: 10.1038/nrm2455 18628784

[B92] Shea A. A. Heffron C. L. Grieco J. P. Roberts P. C. Schmelz E. M. (2023). Obesity modulates the cellular and molecular microenvironment in the peritoneal cavity: implication for ovarian cancer risk. Front. Immunol. 14, 1323399. doi: 10.3389/fimmu.2023.1323399 38264656 PMC10803595

[B93] Shi Q. Shen Q. Liu Y. Shi Y. Huang W. Wang X.. (2022). Increased glucose metabolism in TAMs fuels O-GlcNAcylation of lysosomal Cathepsin B to promote cancer metastasis and chemoresistance. Cancer Cell 40, 1207–1222.e10. doi: 10.1016/j.ccell.2022.08.012 36084651

[B94] Shi Y. Zheng W. Yang K. Harris K. G. Ni K. Xue L.. (2020). Intratumoral accumulation of gut microbiota facilitates CD47-based immunotherapy via STING signaling. J. Exp. Med. 217, e20192282. doi: 10.1084/jem.20192282 32142585 PMC7201921

[B95] Shibata N. Kunisawa J. Kiyono H. (2017). Dietary and microbial metabolites in the regulation of host immunity. Front. Microbiol. 8, 2171. doi: 10.3389/fmicb.2017.02171 29163449 PMC5681998

[B96] Siegel R. L. Miller K. D. Wagle N. S. Jemal A. (2023). Cancer statistic. CA Cancer J. Clin. 73, 17–48. doi: 10.3322/caac.21763 36633525

[B97] Sipos A. Ujlaki G. Mikó E. Maka E. Szabó J. Uray K.. (2021). The role of the microbiome in ovarian cancer: mechanistic insights into oncobiosis and to bacterial metabolite signaling. Mol. Med. 27, 33. doi: 10.1186/s10020-021-00295-2 33794773 PMC8017782

[B98] Somri-Gannam L. Meisel-Sharon S. Hantisteanu S. Groisman G. Limonad O. Hallak M.. (2020). IGF1R axis inhibition restores dendritic cell antitumor response in ovarian cancer. Transl. Oncol. 13, 100790. doi: 10.1016/j.tranon.2020.100790 32428851 PMC7232112

[B99] Sun N. K. Huang S. L. Chang T. C. Chao C. C. (2018). TLR4 and NFκB signaling is critical for taxol resistance in ovarian carcinoma cells. J. Cell Physiol. 233, 2489–2501. doi: 10.1002/jcp.v233.3 28771725

[B100] Tan W. Duong M. T. Zuo C. Qin Y. Zhang Y. Guo Y.. (2022). Targeting of pancreatic cancer cells and stromal cells using engineered oncolytic Salmonella typhimurium. Mol. Ther. 30, 662–671. doi: 10.1016/j.ymthe.2021.08.023 34400328 PMC8821930

[B101] Ternes D. Tsenkova M. Pozdeev V. I. Meyers M. Koncina E. Atatri S.. (2022). The gut microbial metabolite formate exacerbates colorectal cancer progression. Nat. Metab. 4, 458–475. doi: 10.1038/s42255-022-00558-0 35437333 PMC9046088

[B102] Thamm D. H. Kurzman I. D. King I. Li Z. Sznol M. Dubielzig R. R.. (2005). Systemic administration of an attenuated, tumor-targeting Salmonella typhimurium to dogs with spontaneous neoplasia: phase I evaluation. Clin. Cancer Res. 11, 4827–4834. doi: 10.1158/1078-0432.CCR-04-2510 16000580

[B103] Thursby E. Juge N. (2017). Introduction to the human gut microbiota. Biochem. J. 474, 1823–1836. doi: 10.1042/BCJ20160510 28512250 PMC5433529

[B104] Tonetti C. R. de Souza-Araújo C. N. Yoshida A. da Silva R. F. Alves P. C. M. Mazzola T. N.. (2021). Ovarian cancer-associated ascites have high proportions of cytokine-responsive CD56bright NK cells. Cells 10, 1702. doi: 10.3390/cells10071702 34359872 PMC8306021

[B105] Tong J. Zhang X. Fan Y. Chen L. Ma X. Yu H.. (2020). Changes of intestinal microbiota in ovarian cancer patients treated with surgery and chemotherapy. Cancer Manag Res. 12, 8125–8135. doi: 10.2147/CMAR.S265205 32982410 PMC7494227

[B106] Torre L. A. Trabert B. DeSantis C. E. Miller K. D. Samimi G. Runowicz C. D.. (2018). Ovarian cancer statistic. CA Cancer J. Clin. 68, 284–296. doi: 10.3322/caac.21456 29809280 PMC6621554

[B107] Uribe-Herranz M. Bittinger K. Rafail S. Guedan S. Pierini S. Tanes C.. (2018). Gut microbiota modulates adoptive cell therapy via CD8α dendritic cells and IL-12. JCI Insight 3, e94952. doi: 10.1172/jci.insight.94952 29467322 PMC5916241

[B108] Ursin R. L. Dhakal S. Liu H. Jayaraman S. Park H. S. Powell H. R.. (2022). Greater breadth of vaccine-induced immunity in females than males is mediated by increased antibody diversity in germinal center B cells. mBio 13, e0183922. doi: 10.1128/mbio.01839-22 35856618 PMC9426573

[B109] van de Wijgert J. H. Borgdorff H. Verhelst R. Crucitti T. Francis S. Verstraelen H.. (2014). The vaginal microbiota: what have we learned after a decade of molecular characterization? PloS One 9, e105998. doi: 10.1371/journal.pone.0105998 25148517 PMC4141851

[B110] van Teijlingen N. H. Helgers L. C. Zijlstra-Willems E. M. van Hamme J. L. Ribeiro C. M. S. Strijbis K.. (2020). Vaginal dysbiosis associated-bacteria Megasphaera elsdenii and Prevotella timonensis induce immune activation via dendritic cells. J. Reprod. Immunol. 138, 103085. doi: 10.1016/j.jri.2020.103085 32004804

[B111] Verdegaal E. M. E. Santegoets S. J. Welters M. J. P. de Bruin L. Visser M. van der Minne C. E.. (2023). Timed adoptive T cell transfer during chemotherapy in patients with recurrent platinum-sensitive epithelial ovarian cancer. J. Immunother. Cancer 11, e007697. doi: 10.1136/jitc-2023-007697 37949617 PMC10649798

[B112] Verstraelen H. Vilchez-Vargas R. Desimpel F. Jauregui R. Vankeirsbilck N. Weyers S.. (2016). Characterisation of the human uterine microbiome in non-pregnant women through deep sequencing of the V1-2 region of the 16S rRNA gene. PeerJ 4, e1602. doi: 10.7717/peerj.1602 26823997 PMC4730988

[B113] Viel S. Marçais A. Guimaraes F. S. Loftus R. Rabilloud J. Grau M.. (2016). TGF-β inhibits the activation and functions of NK cells by repressing the mTOR pathway. Sci. Signal 9, ra19. doi: 10.1126/scisignal.aad1884 26884601

[B114] Walther-António M. R. Chen J. Multinu F. Hokenstad A. Distad T. J. Cheek E. H.. (2016). Potential contribution of the uterine microbiome in the development of endometrial cancer. Genome Med. 8, 122. doi: 10.1186/s13073-016-0368-y 27884207 PMC5123330

[B115] Wanderley C. W. Colón D. F. Luiz J. P. M. Oliveira F. F. Viacava P. R. Leite C. A.. (2018). Paclitaxel reduces tumor growth by reprogramming tumor-associated macrophages to an M1 profile in a TLR4-dependent manner. Cancer Res. 78, 5891–5900. doi: 10.1158/0008-5472.CAN-17-3480 30104241

[B116] Wang D. Li L. Zhang Y. Ye K. (2024a). Lipopolysaccharide-educated cancer-associated fibroblasts facilitate Malignant progression of ovarian cancer cells via the NF-kB/IL-6/JAK2 signal transduction. Mol. Biotechnol. doi: 10.1007/s12033-024-01055-3 38305842

[B117] Wang N. Liang H. Zen K. (2014). Molecular mechanisms that influence the macrophage m1-m2 polarization balance. Front. Immunol. 5, 614. doi: 10.3389/fimmu.2014.00614 25506346 PMC4246889

[B118] Wang Z. Qin X. Hu D. Huang J. Guo E. Xiao R.. (2022b). Akkermansia supplementation reverses the tumor-promoting effect of the fecal microbiota transplantation in ovarian cancer. Cell Rep. 41, 111890. doi: 10.1016/j.celrep.2022.111890 36577369

[B119] Wang H. Rong X. Zhao G. Zhou Y. Xiao Y. Ma D.. (2022a). The microbial metabolite trimethylamine N-oxide promotes antitumor immunity in triple-negative breast cancer. Cell Metab. 34, 581–594.e8. doi: 10.1016/j.cmet.2022.02.010 35278352

[B120] Wang M. Y. Sang L. X. Sun S. Y. (2024b). Gut microbiota and female health. World J. Gastroenterol. 30, 1655–1662. doi: 10.3748/wjg.v30.i12.1655 38617735 PMC11008377

[B121] Wang Q. Zhao L. Han L. Fu G. Tuo X. Ma S.. (2020). The differential distribution of bacteria between cancerous and noncancerous ovarian tissues in *situ* . J. Ovarian Res. 13, 8. doi: 10.1186/s13048-019-0603-4 31954395 PMC6969417

[B122] Wang X. Zheng Y. Chen X. Peng C. Zhou S. Shen S.. (2023). 2bRAD-M reveals the difference in microbial distribution between cancerous and benign ovarian tissues. Front. Microbiol. 14, 1231354. doi: 10.3389/fmicb.2023.1231354 37692387 PMC10484612

[B123] Wensel C. R. Pluznick J. L. Salzberg S. L. Sears C. L. (2022). Next-generation sequencing: insights to advance clinical investigations of the microbiome. J. Clin. Invest. 132, e154944. doi: 10.1172/JCI154944 35362479 PMC8970668

[B124] Wu S. Y. Fu T. Jiang Y. Z. Shao Z. M. (2020). Natural killer cells in cancer biology and therapy. Mol. Cancer 19, 120. doi: 10.1186/s12943-020-01238-x 32762681 PMC7409673

[B125] Wu L. Qiao L. Zhang S. Qiu J. Du Z. Sun Y.. (2024). Dual-engineered macrophage-microbe encapsulation for metastasis immunotherapy. Adv. Mater. 36, e2406140. doi: 10.1002/adma.202406140 39023382

[B126] Xie S. Zhang P. Zhang Z. Liu Y. Chen M. Li S.. (2021). Bacterial navigation for tumor targeting and photothermally-triggered bacterial ghost transformation for spatiotemporal drug release. Acta Biomater 131, 172–184. doi: 10.1016/j.actbio.2021.06.030 34171461

[B127] Xu S. Liu Z. Lv M. Chen Y. Liu Y. (2019). Intestinal dysbiosis promotes epithelial-mesenchymal transition by activating tumor-associated macrophages in ovarian cancer. Pathog. Dis. 77, ftz019. doi: 10.1093/femspd/ftz019 30916767

[B128] Xu H. Piao L. Wu Y. Liu X. (2022). IFN-γ enhances the antitumor activity of attenuated salmonella-mediated cancer immunotherapy by increasing M1 macrophage and CD4 and CD8 T cell counts and decreasing neutrophil counts. Front. Bioeng Biotechnol. 10, 996055. doi: 10.3389/fbioe.2022.996055 36246355 PMC9556780

[B129] Yan C. Li K. Meng F. Chen L. Zhao J. Zhang Z.. (2023). Integrated immunogenomic analysis of single-cell and bulk tissue transcriptome profiling unravels a macrophage activation paradigm associated with immunologically and clinically distinct behaviors in ovarian cancer. J. Adv. Res. 44, 149–160. doi: 10.1016/j.jare.2022.04.006 36725186 PMC9936412

[B130] Yang Y. Wang Y. Zeng F. Chen Y. Chen Z. Yan F. (2024). Ultrasound-visible engineered bacteria for tumor chemo-immunotherapy. Cell Rep. Med. 5, 101512. doi: 10.1016/j.xcrm.2024.101512 38640931 PMC11148858

[B131] Yao Y. Li J. Li P. Wang D. Bao W. Xiao Y.. (2022). Bacterially synthesized tellurium nanorods for elimination of advanced Malignant tumor by photothermal immunotherapy. Small 18, e2105716. doi: 10.1002/smll.202105716 34889048

[B132] Yu B. Liu C. Proll S. C. Manhardt E. Liang S. Srinivasan S.. (2024). Identification of fallopian tube microbiota and its association with ovarian cancer. Elife 12, RP89830. doi: 10.7554/eLife.89830.3.sa3 38451065 PMC10942644

[B133] Yuan X. Zhang J. Li D. Mao Y. Mo F. Du W.. (2017). Prognostic significance of tumor-associated macrophages in ovarian cancer: A meta-analysis. Gynecol Oncol. 147, 181–187. doi: 10.1016/j.ygyno.2017.07.007 28698008

[B134] Zhou B. Sun C. Huang J. Xia M. Guo E. Li N.. (2019). The biodiversity composition of microbiome in ovarian carcinoma patients. Sci. Rep. 9, 1691. doi: 10.1038/s41598-018-38031-2 30737418 PMC6368644

[B135] Zhou L. Zhao T. Zhang R. Chen C. Li J. (2024). New insights into the role of macrophages in cancer immunotherapy. Front. Immunol. 15, 1381225. doi: 10.3389/fimmu.2024.1381225 38605951 PMC11007015

[B136] Zhu Z. Cai J. Hou W. Xu K. Wu X. Song Y.. (2023b). Microbiome and spatially resolved metabolomics analysis reveal the anticancer role of gut Akkermansia muciniphila by crosstalk with intratumoral microbiota and reprogramming tumoral metabolism in mice. Gut Microbes 15, 2166700. doi: 10.1080/19490976.2023.2166700 36740846 PMC9904296

[B137] Zhu X. Li K. Liu G. Wu R. Zhang Y. Wang S.. (2023a). Microbial metabolite butyrate promotes anti-PD-1 antitumor efficacy by modulating T cell receptor signaling of cytotoxic CD8 T cell. Gut Microbes 15, 2249143. doi: 10.1080/19490976.2023.2249143 37635362 PMC10464552

[B138] Zhu B. Tao Z. Edupuganti L. Serrano M. G. Buck G. A. (2022). Roles of the microbiota of the female reproductive tract in gynecological and reproductive health. Microbiol. Mol. Biol. Rev. 86, e0018121. doi: 10.1128/mmbr.00181-21 36222685 PMC9769908

[B139] Zorea J. Motro Y. Mazor R. D. Carmi Y. K. Shulman Z. Mahajna J.. (2023). TRAF3 suppression encourages B cell recruitment and prolongs survival of microbiome-intact mice with ovarian cancer. J. Exp. Clin. Cancer Res. 42, 107. doi: 10.1186/s13046-023-02680-7 37121997 PMC10150478

